# From Molecular Signal Activation to Locomotion: An Integrated, Multiscale Analysis of Cell Motility on Defined Matrices

**DOI:** 10.1371/journal.pone.0018423

**Published:** 2011-03-31

**Authors:** Amit Pathak, Sanjay Kumar

**Affiliations:** Department of Bioengineering, University of California, Berkeley, California, United States of America; University of Colorado, Boulder, United States of America

## Abstract

The adhesion, mechanics, and motility of eukaryotic cells are highly sensitive to the ligand density and stiffness of the extracellular matrix (ECM). This relationship bears profound implications for stem cell engineering, tumor invasion and metastasis. Yet, our quantitative understanding of how ECM biophysical properties, mechanotransductive signals, and assembly of contractile and adhesive structures collude to control these cell behaviors remains extremely limited. Here we present a novel multiscale model of cell migration on ECMs of defined biophysical properties that integrates local activation of biochemical signals with adhesion and force generation at the cell-ECM interface. We capture the mechanosensitivity of individual cellular components by dynamically coupling ECM properties to the activation of Rho and Rac GTPases in specific portions of the cell with actomyosin contractility, cell-ECM adhesion bond formation and rupture, and process extension and retraction. We show that our framework is capable of recreating key experimentally-observed features of the relationship between cell migration and ECM biophysical properties. In particular, our model predicts for the first time recently reported transitions from filopodial to “stick-slip” to gliding motility on ECMs of increasing stiffness, previously observed dependences of migration speed on ECM stiffness and ligand density, and high-resolution measurements of mechanosensitive protrusion dynamics during cell motility we newly obtained for this study. It also relates the biphasic dependence of cell migration speed on ECM stiffness to the tendency of the cell to polarize. By enabling the investigation of experimentally-inaccessible microscale relationships between mechanotransductive signaling, adhesion, and motility, our model offers new insight into how these factors interact with one another to produce complex migration patterns across a variety of ECM conditions.

## Introduction

The mechanical and geometric properties of the solid-state extracellular matrix (ECM) can profoundly influence cell motility, proliferation, death, and differentiation [1]–[3]. Cells process these biophysical inputs through signaling networks that include integrins and other cell-ECM adhesion receptors, focal adhesion proteins, and Rho family GTPases, which in turn can regulate the assembly and dynamics of the cellular cytoskeleton and direct gene expression [1]. Localized cytoskeletal remodeling enables establishment of cellular polarity, asymmetric generation of traction forces, and ultimately directional, persistent motility.

Cell motility is classically described as a stepwise process that involves protrusion of the leading edge of the cell, stabilization of nascent adhesions, contraction of the cell body, rupture of rear adhesions, and retraction of the trailing edge, which together lead to net translocation of the cell [2], [3]. Importantly, each step in this process requires localized and dynamic formation and breakage of cell-ECM adhesions and generation of traction forces, which are governed by the activation of force-dependent signals in specific portions of the cell. Thus, cell motility is expected to depend on the biophysical properties of the ECM, and a plethora of experimental evidence has now demonstrated that cell motility is highly sensitive to ECM adhesive ligand density and elasticity [4]–[10]; particularly intriguing is the finding that migration speed depends biphasically on ECM adhesivity [7], [9], [10]. In addition, we recently showed that increasing ECM elasticity induces faster motility and strongly regulates the individual steps in migration: human glioma cells cultured on stiff ECMs (>100 kPa) translocate in a smooth, gliding fashion, cells cultured on intermediate-stiffness (10–100 kPa) ECMs translocate in a “stick-slip” fashion with poor coordination between the advance of the leading edge and rupture of the trailing edge, and cells cultured on highly compliant ECMs (<1 kPa) adopt a rounded morphology with unstable adhesions that do not support appreciable motility [8].

While it is widely acknowledged that these spatially- and temporally-coordinated signaling events are critical to motility, progress in this field is limited by a lack of computational models that couple these localized signals to cellular motility and force generation. The vast majority of existing models have either focused on isolated molecular-scale components or modeled the entire cell as a continuum structure without significant molecular detail [4], [9], [11]–[19]. Moreover, comparatively few of these models incorporate the biophysical properties of the ECM. For example, while the compartmentalized cell model [4] establishes a biphasic relationship between migration speed and substrate adhesivity, it does not address potential relationships between ECM stiffness, contractility and protrusion; the traction dynamics model for filopodia [11] provides valuable insights into the mechanosensitivity of protrusive adhesions but omits other components of the motility machinery needed for adhesive maturation and cell translocation.

To build upon these ongoing efforts and strengthen our understanding of the molecular basis of cell-ECM mechanosensing in migration, we developed a novel multiscale mathematical model of cell migration, which dynamically incorporates compartmentalized molecular signaling events with adhesion stabilization and rupture, stress fiber contractility, Rac GTPase-dependent protrusion and stabilization of adhesions, and Rho GTPase-dependent mechanical coordination between front and rear adhesions. This model enables us to probe a wide variety of complex cell-ECM adhesion and migration behaviors, ranging from localized spatio-temportal adhesion dynamics responsible for “stick-slip” migration patterns observed on intermediate-stiffness ECMs to the overall dependence of migration speed on ECM stiffness and ligand density. We also validate the model predictions with new, high-resolution phase contrast imaging of human glioma cells cultured on ECM substrates of defined stiffness.

## Materials and Methods

### Overview of model

**Figure 1:**
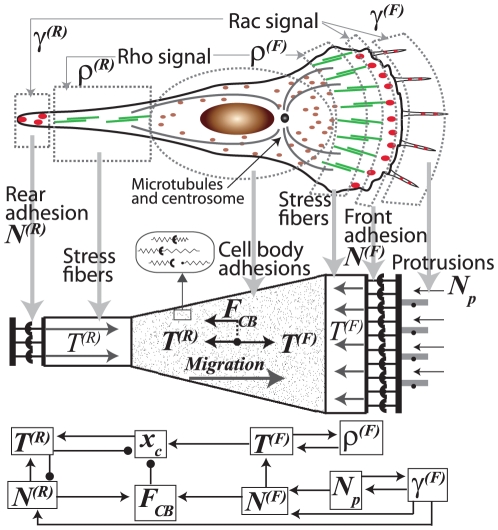
Model components and connectivity. A schematic demonstrating the mapping of a two-dimensional cell into a one-dimensional framework and a flowchart of coupling among time-dependent variables, where arrowheads (→) and bulleted-heads (–•) depict positive and negative feedback mechanisms, respectively.

**Figure 2:**
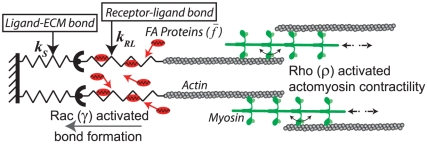
Schematic of interactions between focal adhesions and actomyosin bundles. Here, the adhesive bonds, depicted as springs in series, are strengthened by an influx of adhesion proteins. The contractile machinery, based on relative sliding between actin-myosin filaments, is affected by the deformation rate of the attached adhesion bonds.

We conceptually reduce the cell to a one-dimensional geometry consisting of adhesion clusters with 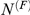 and 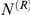 cell-ECM adhesions at the front (leading) and rear (trailing) edges, respectively, interconnected by a set of contractile stress fibers and the cell body (Fig. 1). Our adhesion dynamics model governs stabilization of transient bonds by an influx of adhesion proteins and force-dependent rupture of stable bonds. The resulting coupling between contractile and adhesive components, enforced by mechanical equilibrium, provides the basis for cell-ECM mechanosensing (Fig. 2). Stress fiber contractility, which originates from the relative sliding of actin filaments and myosin heads, i.e. cross-bridge cycling, is strain rate-dependent [20] and regulated by the bond deformation rate of the attached adhesions. The combined multiscale model calculates the integrated cell migration response, a result of coupled processes involving polarization, protrusion, contraction, translocation, and retraction.

The front adhesion grows according to the protrusion/adhesion dynamics model, which includes Rac-dependent formation of lamellipodial protrusions, stabilization of nascent adhesions (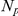) regulated by ECM properties, and their dissociation by membrane ruffles. The frontward polarity is implemented by allowing Rho (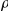) and Rac (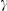) activation levels in the front to grow according to the protrusion and contractility dynamics models. At the rear, restricting Rac activation to a minimum value 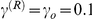 curtails trailing-edge protrusions altogether, and setting Rho activation to its maximum 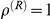 facilitates maximal contractile force generation. The frontward polarization of the cell ensures that the inward contractile force acting at the front adhesions is higher than at the rear adhesions, i.e. 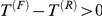, which is responsible for net forward translocation of the cell body (Fig. 1). In addition, adhesions distributed across the cell-ECM interface exert an effective resistive force on the cell body 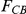, which opposes cell translocation. By incorporating transient as well as stable adhesions dispersed over the cell body, 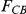 captures the effects of adhesion complexes not included in the front and rear adhesion clusters defined in our 1D construct (Fig. 1), and balances the traction force asymmetry in the front and rear as described later in greater detail (Eq. 10).

The net forward propulsive force, which is due to frontward polarization driven by lamellipodial protrusions (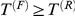), dictates the rate of translocation of the cell centroid, 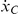, based on force equilibrium at the cell level (Fig. 1; Eqs. 9–11). The adhesions at the trailing edge cumulatively rupture due to both the increasing contractile force, 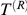, and the reaction force caused by forward translocation of the body, 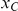. As these cycles of formation, retraction and re-initiation of the rear adhesion clusters are repeated, the cell moves forward in a step-wise manner captured by several time-dependent variables – 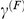, 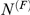, 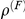, 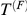, 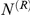, 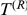 and 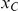 – subject to the dynamical system presented below (Fig. 1, Eqs. 3–9; see Text S1 for a complete glossary of model variables and constants).

### Adhesion growth and rupture dynamics

Several previous models have imposed *ad hoc* energy-based criteria to compel force-dependent adhesion growth [4], [17], [21]. At the molecular scale, this mechanosensitivity originates from force-dependent conformational and binding properties of specific proteins in the ECM and adhesions [22]. Here we integrate these two descriptions and subject adhesion dynamics to two competing force-dependent inputs that favor growth and rupture of the adhesion. Rac activation promotes formation of transient adhesions, i.e., focal complexes, which are stabilized and transformed into stable focal adhesions (FAs) by an influx of mechanosensitive proteins. Conversely, as these contacts are loaded beyond the adhesive capacity of the ECM, they dissociate according to a bond rupture dynamics model based on an energy criterion similar to one previously described elsewhere [21]. We envision cell-ECM adhesion as receptor-ligand and ligand-ECM bonds in series, defined by linear Hookean springs of elasticities 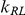 and 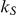, respectively (Fig. 2). Increased bond stretch exposes cryptic sites to which FA proteins bind, which in turn strengthens the transient bonds and reduces the turnover rate of the cluster. In this way, the model begins to incorporate alterations in molecular conformation. Thus, the concentration of FA proteins accumulated per receptor-ligand bond is 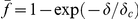, where the argument in the exponential is the bond stretch 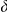 normalized by a critical bond stretch 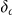. For an adhesion cluster of 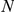 bonds supporting a net force 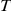, we can calculate the bond stretch by 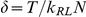. The size of an adhesion cluster is governed by two rate-dependent processes working in tandem. First, an association rate due to clustering of receptors and FA proteins is defined as
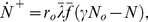
(1)


where 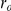 is a reference rate constant for receptor clustering, and 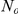 is the maximum allowable receptors in an adhesion cluster (henceforth, a dotted parameter will denote its first time-derivative). The number of active receptors depends on the Rac activation level 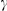, which itself is regulated by lamellipodial protrusions as discussed below. Cell-ECM receptor-ligand interactions are governed by the density of ligand proteins on the surface, 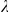, defined here as ECM adhesivity 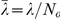. These transient receptor-bonds are stabilized by 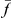 as defined above.

Second, a dissociation rate is determined by the rupture dynamics of the stable adhesion bonds from excessive applied force:
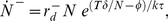
(2)


where 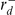 is a rate constant for bond dissociation; 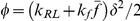 is the stored potential energy per bond with an additional spring constant contribution 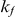 per unit concentration of FA proteins that stabilize the adhesion cluster by an additional potential energy contribution; 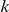 is the Boltzmann constant, and 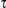 the absolute temperature. In our model, the dissociation rate increases exponentially with 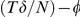, which is the balance of external work due to the tensile force (*T*) exerted by the attached contractile stress fibers and the stored potential energy per bond [21]. For all subsequent calculations, we use 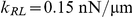, 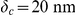, yielding a receptor-ligand bond strength of 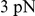 consistent with [23], 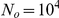, as in [4], and 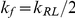 (our assumption for the calculations). We choose rate constants 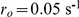 and 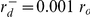, adapted from [4], which can be recalibrated for different conditions. The adhesion dynamics model developed thus far suffices for stationary adhesions unaffected by protrusion-related growth in a polarized cell. The following section addresses protrusion dynamics.

### Protrusion stabilization and Rac activation dynamics

Rac-dependent actin polymerization at the leading edge of the cell regulates the formation of lamellipodial protrusions that adhere to the substrate and produce a net propulsive force [2], [3], [11]. However, high Rac activation also causes membrane ruffles that can destabilize the nascent adhesions at the lamellar base [24]. The energy associated with ruffle advancement is dissipated by the transient loading and failure of adhesions between membrane protrusions and ECM ligands. We capture these coupled processes by incorporating two competing mechanisms that define formation and dissociation rates of adhesions. We can describe the net growth rate of protrusion adhesions (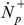) as:
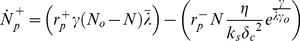
(3)


Here, the first term represents the forward rate of formation of lamellipodial transient adhesions governed by the Rac activation level 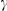, ECM adhesivity 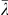, and a constant intrinsic rate of adhesion formation at the filopodia estimated to be 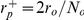. The second term for the dissociation (backward) rate of nascent adhesions is proportional to the following: (1) A rate constant 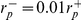; (2) The size of the leading-edge adhesions *N*; (3) The energy associated with membrane ruffling 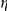 normalized by a ECM stiffness-dependent bond rupture energy 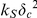; and (4) A dissociation rate that exponentially increases with Rac activation level 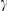 normalized by the product of the minimum allowable Rac activation level 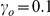 and the surface adhesivity 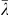. We estimated the value of 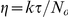 based on the reference parameters used in our calculations.

In addition, a positive feedback loop between Rac activation and rate of protrusion stabilization [2], [25] leads to a rate of Rac activation 
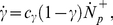
(4)


with a proportionality constant 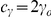 per bond.

The forward and backward rates for the adhesion size due to receptor clustering, bond rupture, and protrusions, (Eqs. 1–3) are combined to yield a rate of adhesion growth of: 
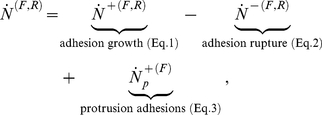
(5)


where superscripts *F* and *R* denote front and rear as before.

### Actomyosin contractility and Rho signaling

Contractile actin bundles (e.g. stress fibers) consist of antiparallel arrays of F-actin and a variety of accessory proteins interleaved with filaments of nonmuscle myosin II, whose motor function both directly underlies cell contractility and is regulated by Rho activated signaling through adhesions [2]. To express this connection between contractility and adhesion, we assume that the contractile force generated by a stress fiber is proportional to the size of its attached adhesion cluster (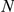) and the activation level of the Rho signal (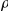) – defined here as 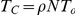, with 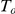 as the maximum contractile force generated per actomyosin motor. It has also been previously established that the strain-dependent cross-bridge cycling between actomyosin filaments governs the amount of tension sustained by the stress fiber [20], [26]. ECM rigidity governs the deformation of cell-ECM adhesions, which disrupts the strain-dependent cross-bridge cycling of the attached actomyosin assembly; as a result, the force sustained by the stress fiber (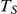) falls below the generated contractile force (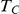). This mechanism is formulated as 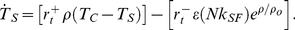
(6)


Here, the first term governs the growth of contractile force sustained by the stress fibers (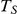) proportional to the force generated (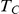) and Rho activation level (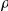). The second term is responsible for the loss of contractile force due to the deformation of cell-ECM bonds (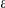) that oppose actomyosin contractility by effectively relaxing the stress fibers, and it includes an exponential factor of normalized Rho activation level (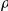) that represents the excessive cross-bridge cycling due to rapid activation of Rho signaling, which must be equilibrated by ECM reactionary forces. The spring constant of the stress fibers is estimated by 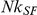, where 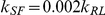 per adhesion bond. At mechanical equilibrium, the net tension in the stress fiber 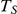 is equal to the force supported by the adhesion cluster; thus 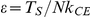, where 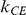 is the equivalent stiffness of a cell-ECM bond, calculated as 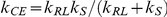. In our calculations, we chose 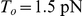 [26], the normalization constant for Rho activation 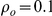, and rate constants 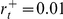 and 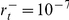.

Rho signaling itself is known to be regulated by the contractile forces exerted by stress fibers [27], which is expressed here as
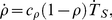
(7)


with a proportionality constant 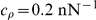.

It is also known that the contractile machinery and Rho signaling are maximally active at the trailing edge while the development of contractility at the leading edge follows more complex dynamics [27]. Hence, we assume that the aforementioned dynamical system for force 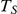 and Rho signal 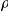 (Eqs. 6–7) only applies for the leading edge. On the other hand, the force exerted by the trailing edge is a combination of the maximum contractile force generated by actin-myosin machinery (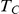) and an additional pull force due to forward migration of the cell body while the trailing edge adhesions remain intact. Based on these observations, the net forces exerted by the stress fibers in the front and rear are written respectively as:
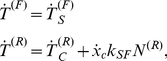
(8)


### Migration of the cell body

As discussed earlier, the rate of translocation of the cell centroid (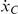) results in part from the frontward polarization of the cell. The force equilibrium at the cell level (as described schematically in Fig. 1) can be written as 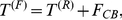
(9)


where frontward forces (left side) are always balanced by the rearward forces (right side), which includes both contractile forces applied to rear adhesions and an effective “drag” force imposed by central adhesions, which must be disassembled in order for the cell to translocate. This cell-body drag force (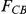) is calculated as
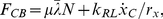
(10)


Here, the first term describes the resistive force absorbed by the transient receptor-ligand bonds being formed and turned over continuously across the entire cell body, formulated as the product of the number of cell-body adhesions (estimated as the square of the number of adhesions in the front, i.e. 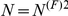), a proportionality constant 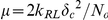, and ECM adhesivity (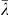). The second term represents the force exerted by the stable and localized cell-ECM adhesions dispersed over the cell body that have not been accounted for in the front and rear adhesion clusters. These stable cell-body adhesions deform during forward migration of the cell body and exert a force proportional to the rate of displacement of the cell body, with a rate constant 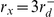. It should be noted that our definition of the cell-body resistive force (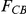) is distinct from the conventional definition of the viscous drag force that opposes the relative motion between two surfaces, which would not incorporate active receptor-ligand bonds that are formed, stabilized, turned over, and ruptured at all times in this actively evolving cell-ECM system. We anticipate that a purely viscous drag force at the cell-ECM interface would be negligible compared to the cell-body resistive force (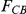) as defined above.

By combining Eqs. 9 and 10, the rate of cell displacement (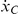) can be formulated as
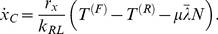
(11)


### Experimental validation of model predictions

**Figure 3:**
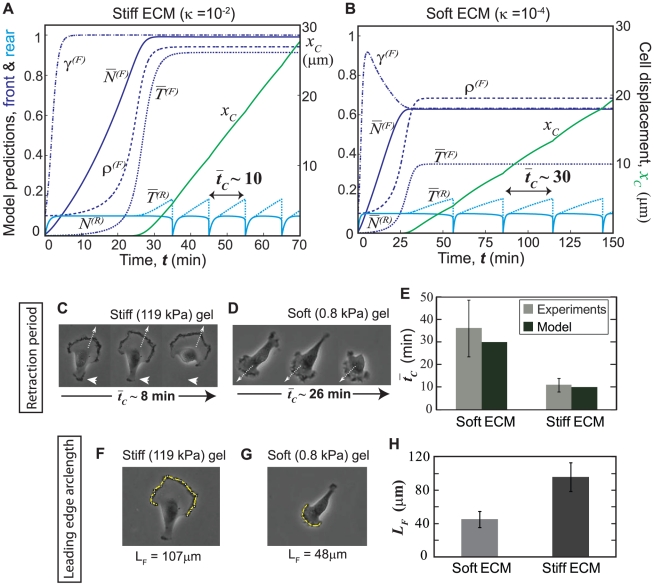
Migration cycles and ECM stiffness: Model predictions and experimental results. Model calculations of “stick-slip” migration phenotype in terms of normalized values of adhesion size (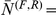 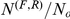) and tension generated by the attached stress fibers (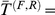 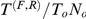) at front and rear adhesions to demonstrate: (a) faster migration with smaller retraction period 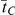 on stiff ECMs, and (b) slower migration with higher 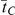 on soft ECMs. Experimental quantification of retraction period for a cell migrating on (c) a stiff 119 kPa gel, and (d) soft 0.8 kPa gel. (e) Comparison of 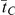 predicted by the model and measured in the experiments. Membrane profiles at the leading edge of the U373-MG glioma cells migrating on polyacrylamide gels of (f) 119 kPa and (g) 0.8 kPa stiffness. (h) Comparison of archlengths *L_F_* of the leading edge observed in glioma cells migrating on stiff (119 kPa) and soft (0.8 kPa) gels. Error bars represent the SD about the means of 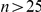 retraction cycles, 1–3 retractions per cell.

We performed time-lapse phase-contrast imaging of U373 MG human glioma cells migrating randomly on fibronectin-coated polyacrylamide gels as described previously [8] but at a much higher frame capture rate than before (here, every 2 min). These videos enabled us to measure the amount of time needed for the trailing edge of the cell to retract completely starting from a polarized state on highly stiff (119 kPa) and compliant (0.8 kPa) ECMs (Fig. 3C–D; Supplementary Videos S1 and S2). All data represent at least two independent biological replicates (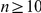 cells), where 20X phase contrast time-lapse images were acquired every 2 min over a 3 h period for at least 5 different fields of view. We then measured the time required for each cell to progress from a fully polarized state with distinct leading and trailing edges to retraction of the trailing edge, which we defined as the retraction period (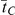), analogous to the computational model. We also measured the arclength of the leading edge by using the segmented line tool in the Image J software (NIH) from the phase contrast videos. We obtained these data for cells on stiff and soft ECMs, which correspond to polyacrylamide gels of Young's Moduli of 119 kPa and 0.8 kPa, respectively [8].

## Results and Discussion

### Cell migration on defined-stiffness ECMs: Model predictions and comparison with experiment

To test the predictive capabilities of our model, we simulated cell migration on ECMs of varying stiffness 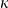 (defined as 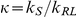, where the cell-ECM bond stiffness 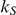 is the varied parameter) and adhesivity 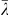. The ECM stiffness parameter in the model, 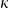, represents a molecular-scale spring constant, which can be translated into a Young's modulus using basic assumptions of linear elasticity. One simple relation (modified from [17]) that can be used to make this connection is: 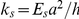, where 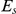 is the Young's modulus of a material with effective spring constant 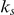, 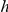 (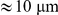) is the estimated thickness of the gel that experiences reaction forces transmitted via cell-ECM adhesions, and 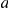 (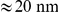) is the standard size of the adhesions [28]. Using this formula we estimate that the bulk stiffness of the ECMs ranges from approximately 0.4 kPa to 400 kPa as ECM stiffness ratio in the model (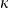) is varied from 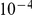 to 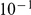.

We first simulated cell migration on ECMs of varying 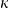 for a constant ECM adhesivity 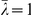, and plotted the temporal evolution of Rho and Rac signaling in the front as well as adhesion and contractility dynamics in both the front and rear of the cell (Fig. 3A–B and Fig. S1). In these plots, Rho and Rac activation at any given time point are calculated from time-dependent actomyosin contractile force and adhesion size as described in differential equations 4 and 7. On stiff ECMs, more protrusive adhesions are stabilized due to higher Rho and Rac activation levels, leading to maximal stress fiber contractility, and rear adhesions progress through the initiation-rupture cycle rapidly. Both of these promote higher migration speeds (Fig. 3A). Conversely, soft ECMs require a longer characteristic feedback time, which slows development of contractile forces and stabilization of adhesions due to large bond deformations within adhesions and lower Rho and Rac, activation. Thus, the overall effect of low ECM stiffness is both to reduce the ability to advance the leading edge via protrusion, and to reduce the rate of development of the force exerted by the trailing edge. This in turn facilitates adhesive rupture, resulting in longer times between retractions (Fig. 3B). The frontward polarity of the cell ensures that the total contractile force generated in the front (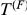) is higher than the contractile force in the rear (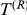), consistent with the asymmetric distribution of forces in the traction force microscopy maps of migrating cells [29]. That said, the force concentration is higher in the rear, i.e. 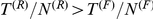, while 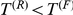, because 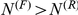, which causes faster adhesion rupture in the rear, retraction of the trailing edge, and forward translocation of the cell.

These cycles of rear edge retraction and re-initiation events produce a “stick-slip” migration phenotype, which we can quantitatively characterize by calculating the retraction period (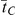), the cycle time for rear edge retractions. Our model predicts that 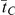 should increase threefold as ECM stiffness is reduced by three orders of magnitude (Figs. 3A–B), which is grossly consistent with our previously reported findings [8]. However, to validate our model predictions more rigorously, we performed new time-lapse migration studies in which we tracked cell migration on soft and stiff ECMs at a much higher temporal resolution, which in turn enabled us to quantify and statistically analyze trailing-edge protrusion-retraction cycles. We then used these data to measure the experimental protrusion-retraction cycle times, and found that this experimental 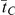 varies with ECM stiffness almost exactly as predicted by the model (Fig. 3C–E). We also found that on softer ECMs, cells traveled shorter distances and took longer times between rear edge retractions (Fig. 3B). This, together with the suppression of protrusion stabilization observed at the leading edge, resulted in slower overall migration speeds. The displacement trajectory on stiff ECMs is smoother (Fig. 3A) than on soft ECMs (Fig. 3B), in contrast to the more prominent “stick-slip” migration phenotype observed on soft ECMs.

 Furthermore, the model predicts that the density of adhesions should fall by approximately 40% as one goes from a stiff ECM to a soft ECM (compare the plots for 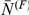 in Figs. 3A–B). This model prediction for adhesion density can be tested experimentally by measuring focal adhesion area using immunofluorescence and/or fluorescently-tagged focal adhesion proteins; however, it is difficult to quantify these accurately, particularly for smallest and most nascent adhesions likely to be most important for establishing lamellipodial contacts. Assuming that the number of cell-ECM adhesions in the lamellar region is proportional to the spreading profile of the membrane at the leading edge, we measured the arclength, 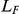, of the membrane along the leading edge of the migrating cell as described above. These measurements revealed that 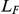 falls by approximately 50% on soft ECM as compared to the stiff ECM (Fig. 3H), which agrees well with the model prediction.

### Mechanosensitive cellular components respond to ECM stiffness and adhesivity

**Figure 4:**
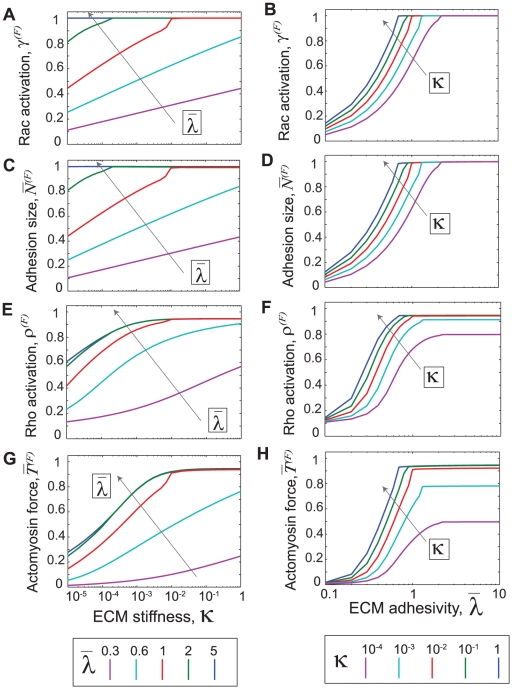
Response of specific model components to ECM stiffness and adhesivity. Each family of curves represents the dependence of a specific model component (shown to the left of each row) on ECM stiffness 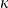 (left column) and ECM adhesivity 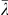 (right column) when one ECM parameter is assigned to a series of discrete values and the other is systematically varied. For example, plots (a–b) depict the dependence of Rac activation 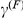 on (a) ECM stiffness 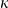 for five different ECM adhesivity values 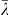, and (b) 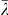 for 5 different values of 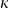. Similarly, the second, third and fourth rows depict the effect of ECM stiffness and adhesivity on: (c)–(d) leading-edge adhesion density 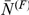; (e)–(f) Rho activation 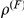; and (g)–(h) leading-edge contractile force 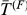.

 The mechanosensitivity of cell migration *in toto* reflects the collective contributions of each mechanosensitive subcellular component. To dissect this relationship more quantitatively, we next investigated how individual model mechanisms, including activation of Rho and Rac GTPases as well as contractility and adhesion dynamics, respond to a wide range of ECM properties and eventually contribute to variations in migration phenotypes. The model predictions and experimental correlations clearly demonstrate the capabilities of the model for two extreme cases of ECM stiffness (Fig. 3). To explore behaviors at intermediate values, we repeated the simulations for a wider range of ECM stiffness and adhesivity values and tracked the steady state levels of Rho and Rac activation signals (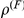, 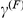), adhesion sizes (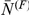) and actin-myosin contractile forces (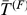) at the leading edge of the cell (Fig. 4).

Rac activation (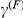) reaches its maximum value at high ECM adhesivity regardless of stiffness (note the plots for 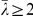 in Fig. 4A), while at lower adhesivity 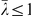 it increases with ECM stiffness; on softer ECMs, Rac activation requires higher adhesivity (Fig. 4B). Consistent with the codependent coupling between Rac activation and adhesion dynamics formulated in our model (Eqs. 1–5), the front adhesion size (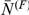) follows the same trend as the Rac activation level (Fig. 4C–D). Note that both Rac activation and adhesion growth are more sensitive to ECM adhesivity than to ECM stiffness; however, stiffer ECMs produce maximal Rac activation and adhesion sizes in each case.

Rho activation evolves to higher levels as ECM stiffness is increased and is largely insensitive to ECM adhesivity (Fig. 4E–F). In our model, the maximum contractile force is limited by the degree of Rho and Rac activation, which in turn contribute to adhesion size. More specifically, while Rac activation dictates adhesion size and thereby limits stress fiber tension per adhesion, Rho activation directly contributes to stress fiber contractility through myosin activation (Eqs. 6–8). Accordingly, stress fiber force increases with both ECM stiffness and adhesivity (Fig. 4G–H). Our prediction that Rho activation and actomyosin forces increase on stiffer ECMs agrees with previous experimental observations [30], [31].

### Variation in migration speed over a wide range of ECM properties

**Figure 5:**
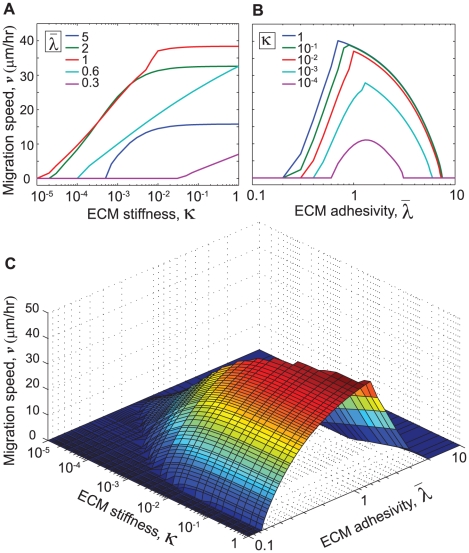
Dependence of migration speed on ECM properties. Average migration speed 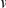 as a function of (a) normalized ECM stiffness 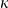 for different values of ECM adhesivity 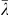; and (b) ECM adhesivity for different values of ECM stiffness. (c) The combined 3D surface plot of migration speed versus ECM stiffness and adhesivity.

During each migration cycle, the cell centroid moves by a distance 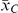 over a time 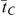. At steady state, the average step migration speed is calculated as 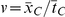. Our model predicts that migration speed increases with ECM stiffness and saturates (Fig. 5A,C), which completely agrees with our earlier experimental observations [8] but has thus far lacked a clear mechanistic basis. Our model supports the notion that faster motility on very stiff ECMs (Fig. 5 A) arises from higher activation levels of Rho and Rac signals (Fig. 4 A,E), and larger adhesions and contractile forces in the front (Fig. 4 C,G) that induce faster detachment in the rear. On the other hand, migration speed has a biphasic dependence on ECM ligand density, as reported previously [4], [6], [9], but how this behavior might change on ECMs of systematically varying stiffness has not been extensively explored. We calculated cell migration speed on ECMs of varying ligand density for different values of ECMs stiffness (Fig. 5B). Consistent with the experimental observations in an earlier report [7], our model predicts that lower ligand-density surfaces require higher ECM elasticities to reach the maximum migration speed, whereas higher ligand-density surfaces enhance migration speed on relatively soft ECMs (Fig. 5A). In our model, low ligand density reduces the forward rate of adhesion stabilization with lower levels of Rac and Rho activation, which leads to an insufficient propulsive force for cell translocation (Fig. 4). Conversely, high ligand density leads to greater adhesive engagement along the cell body, causing an excessive drag force and retarding migration. The net result of these two effects is that migration speed varies biphasically with ECM ligand density. On stiff ECMs, cells develop the maximum allowable levels of adhesion, contractile force, and Rac activation (Fig. 4) such that even extreme values of ligand density can support migration (Fig. 5B,C), whereas soft ECMs yield slower development of adhesions and contractility (Fig. 4C,G). Thus, ECMs of optimum ligand density are required to cause a positive propulsive force and promote migration, and the steepness of this optimum depends strongly on ECM stiffness (note the narrower range of allowable ligand densities on softer ECMs in Fig. 5B). This prediction also echoes an earlier study in which the migration speed of smooth muscle cells was found to increase monotonically with ECM stiffness at low ligand density; migration speed then falls dramatically on tissue culture polystyrene, which is presumably so rigid and adhesive as to produce hyperstable stress fibers and focal adhesions that abolish cycling altogether and trap the cell in a well-spread, non-motile regime [7].

### Intrinsic cell polarity influences the biphasic dependence of migration speed on ECM stiffness

In the preceding simulations, we enforced frontward cell polarity by restricting Rac activation in the rear to 10% of the maximum allowable level (i.e. 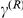 = 0.1), while the allowing protrusion and Rac activation dynamics at the front of the cell to proceed under the governing equations described earlier (Eqs. 3–4). As reported above, this reliably captures the migration phenotype exhibited by U373 MG human glioma cells; however, other cell types may not display the same degree of polarity in their morphology. For example, vascular smooth muscle cells (SMCs) have been reported to display strong adhesions in both front and rear of the cell and to generate multiple lamellipodia [7], and it may be inappropriate to impose the assumptions of our model on these and other weakly-polarized cells. We therefore explored what might happen if we relaxed the requirement of enforced frontward polarity.

Under these conditions, we envision that adhesions and Rac activation in the rear would grow in a mechanosensitive fashion, just as they would at the front of the cell. This can be implemented numerically by replacing the user-defined constant Rac activation level in the rear (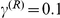) with the protrusion and Rac activation dynamics model implemented in the front of the cell (presented above in Eqs. 3–5). However, some degree of cell polarity is mandatory for cell migration [3], as equal growth of adhesions and contractility in both front and rear would preclude the breaking of symmetry that is required for motility. A modest polarity can be achieved by introducing a polarization factor 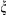 in the adhesion growth and Rac activation model applied in the rear. As this factor grows larger, the more polarized the cell becomes. Thus, similar to the protrusion dynamics model for the front (Eqs. 3–4), the equations for temporal evolution of Rac activation level (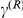) and adhesion size (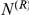) in the rear can be written as
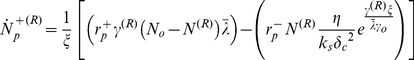
(12)

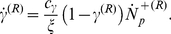
(13)


The forward and backward rates for the adhesion size due to receptor clustering, bond rupture, and protrusions, (Eqs. 1–3 for the front, and Eqs. 1–2 with Eq. 12 for the rear adhesions) are combined to yield a rate of adhesion growth of: 
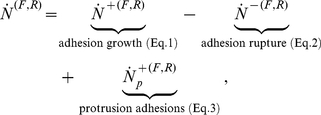
(14)


where superscripts *F* and *R* denote front and rear as before.

Thus, for a polarization factor 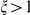, growth of Rac activation and adhesion size in the rear would be slower compared to the front, which would enable the frontward polarity required for cell migration. Conversely, values of 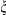 approaching 1 would curtail spatial asymmetry in the cell by strengthening the rear adhesion, which in turn would reduce the net propulsive force, retard the rear edge retraction cycle and slow migration. Note that under these conditions, rear adhesion growth and Rac activation model (Eqs. 12–13) become dependent on ECM stiffness (with the degree of dependence determined by 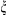), which indicates that the effect of the polarization factor (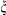) on cell migration should vary with ECM stiffness.

**Figure 6:**
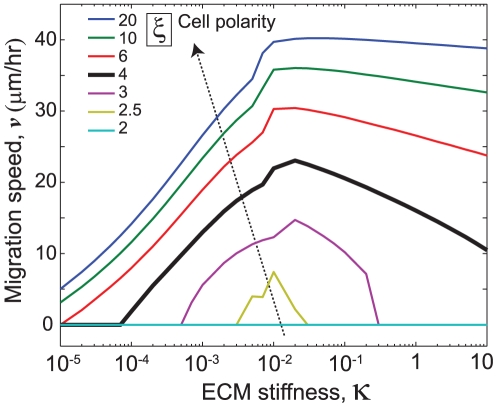
Effect of cell polarity on the relationship between migration speed and ECM stiffness. Average migration speed 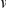 as a function of normalized ECM stiffness 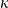 for different values of the polarization factor 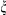. In all cases, ECM adhesivity is kept at a constant level (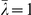).

Our simulations predict that the curtailment in cell polarization induces a biphasic relationship between migration speed and ECM stiffness (Fig. 6). These results, specifically that obtained for 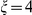, agree with earlier experimental observations for SMCs on ECMs of varying stiffness [7]. The dependence of migration speed on ECM stiffness for high values of the polarization factor (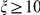) resembles our earlier prediction for highly polarized cells (Fig. 5A) where migration speed increased with ECM stiffness without any biphasic dependence. For lower values of the polarization factor (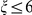), rear adhesions (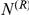) grow faster on stiff ECMs, which reduces cell polarity and slows cell migration.

**Figure 7:**
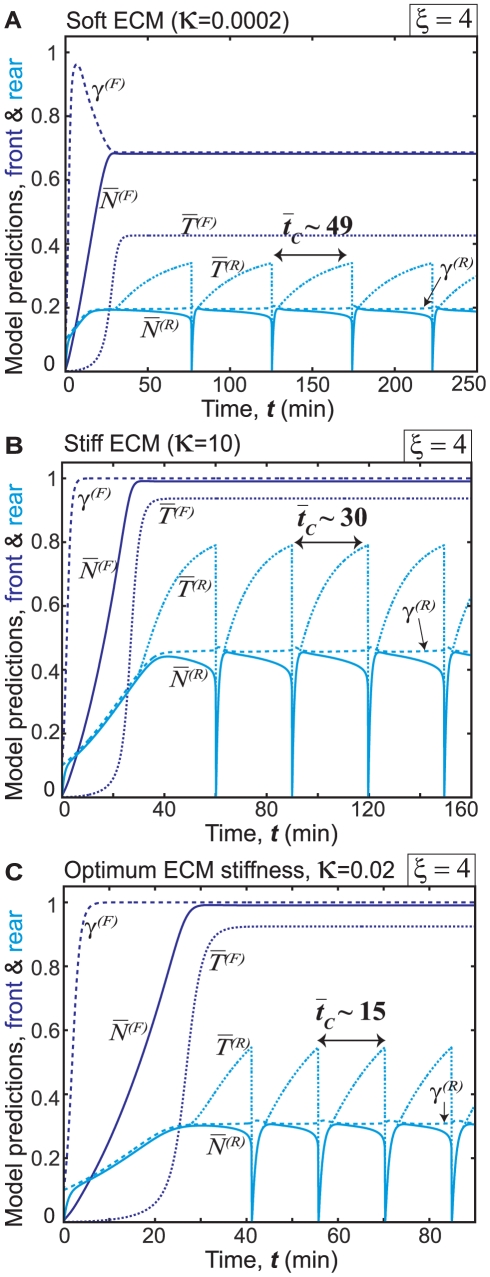
Variation of subcellular mechanisms with ECM stiffness for cells with reduced polarity. Time evolution of normalized values of adhesion size, actomyosin force, and Rac activation levels in the front and rear of the cell (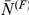, 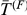, 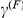, 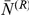, 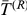, and 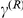), for ECM adhesivity 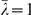 and polarization factor 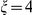, on ECMs of (a) Soft; (b) Stiff; and (c) Intermediate/optimum stiffness. Weak activation of subcellular mechanisms in both front and rear leads to larger retraction period 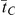 and slower migration on soft ECMs (a). Stiffer ECMs induce enhanced activation of subcellular mechanisms in the rear, stabilize the rear adhesion, increase 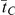 and slow down cell migration (b), while intermediate-stiffness ECMs weaken rear adhesions without significantly altering adhesion at the front of the cell, thus leading to frequent rear edge retractions, i.e. shorter 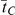, and faster cell migration.

To gain further insight into the subcellular mechanisms that yield a biphasic relationship between migration speed and ECM stiffness for polarization factor 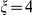, we next examined the relative magnitudes of adhesion size, actomyosin force and Rac activation levels in the front and rear of the cell (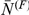, 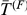, 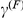, 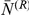, 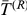, and 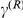), for three different values of ECM stiffness (Fig. 7). On soft ECMs, the adhesive and contractile mechanisms in both front and rear are only weakly activated, thereby yielding insufficient traction forces to drive rear edge retraction and cell migration (Fig. 7A), whatever the cell polarity. Stiff ECMs, conversely, enhance the rates of development of the mechanosensitive subcellular mechanisms for adhesion size, actomyosin contractile force and Rac activation in the rear and yields higher levels of 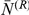, 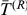, and 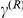 (Fig. 7B). The rear adhesions thus stabilized become harder to rupture and require longer time for rear edge retraction, as demonstrated by higher value of 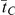 ( = 30 min) (Fig. 7B) compared to the retraction period predicted for polarized cell (Fig. 3A). On intermediate-stiffness ECMs (Fig. 7C), the subcellular mechanisms in the rear weaken without significant change in the front of the cell; this lag between front and rear is attributed to the value of 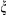 implemented in the rear (Eqs. 12, 13).

The overall finding is that cell types that do not have the inherent tendency to polarize (captured in our model by intermediate values of the polarization factor 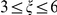), would attain their maximum possible polarity for some optimum values of ECM stiffness, which in turn would expedite rear edge retraction cycles (compare 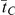 in Fig. 7A–C), yield maximum migration speeds at intermediate values of ECM stiffness, and give rise to a biphasic relationship between cell migration speed and ECM stiffness (Fig. 6). Cells that are strongly polarized would be expected to migrate faster with increasing values of ECM stiffness, as exemplified by the experimental finding that fibroblasts readily migrate from soft ECMs to stiff ECMs but rarely do the reverse [32], [33].

### Conclusions

We have presented a multiscale model of cell migration that for the first time integrates the microscale spatio-temporal dynamics of cell-ECM adhesions, lamellipodial protrusions, stress fiber contractility, activation of mechanotransductive signaling events, and key biophysical properties of the ECM. While previous modeling efforts have provided a valuable starting point for our work [4], [9], [11], [19], we have built upon these efforts by offering molecular mechanistic bases for relationships between the speed and mode of migration and ECM stiffness and ligand density. To summarize: (1) The cell-ECM bond deformation rate regulates the development of intracellular contractility, which in turn governs adhesion rupture dynamics; (2) Rac-dependent protrusion stabilization dynamics, also regulated by ECM stiffness, contributes to cell polarization; (3) Adhesion-dependent contractile forces must rupture rear adhesions to allow faster cell migration. Predictions based on these principles both agree with a wide variety of previous experiments [7], [8] and encompass constellations of ECM properties that had not been explored before.

Our integrated, multiscale modeling framework for cell migration produces several novel predictions and insights that are physiologically relevant for a variety of cell types and ECM conditions in 2D settings, for example: (1) Migration speed is directly related to cycling times between protrusion, adhesion, and retraction; (2) Even on stiff ECMs, insufficient ligand density can reduce cell migration speed (Fig. 5) and place fundamental limits on contractilie force, adhesion size and Rho/Rac activation; (3) The biphasic relationship between ligand density and cell migration speed depends on ECM stiffness, such that softer ECMs allow a narrower range ligand densities optimum for cell migration (Fig. 5B); (4) In highly polarized cells, cell migration speed increases with ECM stiffness and reaches a plateau, while less polarized cells exhibit a biphasic relationship between ECM stiffness and migration speed (Fig. 6; see Fig. 7 for a mechanistic explanation for this prediction). Critically, even though our model focuses in part on the importance of ECM stiffness to cell motility, we do not suggest that this is the only physical regulator of cell motility; indeed, the tendency of the cell to polarize, the adhesivity of the ECM surface, and many other factors contribute strongly to migration.

A potential limitation of this model is the use of a 1D geometry, which may fail to capture some of the complexities of migration in 2D and 3D topologies. Despite this important simplification, model predictions agree surprisingly well with our experimental observations of 2D cell migration, including migration speed and aspects of lamellipodial extension and force generation. Additionally, given the recent discovery that migration in tissue ECMs may more closely resemble 1D migration than 2D migration [34], one could argue that our model should do a *superior* job of simulating 3D migration than one that incorporated 2D details. In future models, it will be interesting to test this hypothesis more directly by explicitly incorporating these topological details. These next-generation models should also offer a good opportunity to include key structural details into the ECM, such as fiber diameter, mesh size, and nonlinear elastic properties, all of which are expected to play key roles in tumor cell invasion [35].

Our model also predicts several relationships which could be tested experimentally in the future, including the dependences of front and rear adhesion sizes, contractile forces, time between migration cycles, and migration speed on a wide range of ECM properties. We also provide a potentially testable mechanistic explanation for why some cell types exhibit a biphasic dependence of migration speed on ECM stiffness that is rooted in the tendency of that cell type to polarize. Experimental validation of these predictions should facilitate even more sophisticated and experimentally informed modeling efforts in the future, which may both lend greater insight into the underlying biological problem and permit simulations of cell migration in more complex microenvironments that better approximate the in vivo setting.

## Supporting Information

Figure S1Model calculations of “stick-slip” migration phenotype in terms of normalized values of adhesion size (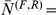 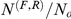) and tension generated by the attached stress fibers (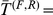 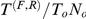) at front and rear adhesions to for ECM stiffness parameter (a) 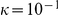, and (b) 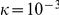. Note that the difference between the retraction period (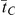) in these two cases is not as wide as between 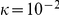 and 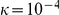, presented in Fig. 3 A, B in the main manuscript. These simulations demonstrate that the cell response, calculated in terms of rear edge retraction period, distance travelled and other time dependent variables (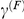, 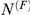, 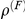, 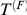, 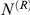, 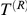), reaches a maximal plateau at around 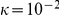 and does not change drastically for higher value of ECM stiffness.(EPS)

Video S1 Phase contrast video of rear edge retraction of a U373-MG cell migrating on a 119 kPa fibronectin-conjugated polyacrylamide ECM.
(AVI)

Video S2 Phase contrast video of rear edge retraction of a U373-MG cell migrating on a 0.8 kPa fibronectin-conjugated polyacrylamide ECM.
(AVI)

Text S1 Glossary of time-dependent variables and model constants.
(DOC)
