# Supplementary material for: From Molecular Signal Activation to Locomotion: An Integrated, Multiscale Analysis of Cell Motility on Defined Matrices
Source: PLoS One. 2011 Mar 31;6(3):e18423. doi: 10.1371/journal.pone.0018423 (PMC3069105; doi:10.1371/journal.pone.0018423)
Supplement: Text S1 — Glossary of time-dependent variables and model constants. (DOC) [file pone.0018423.s004.doc]

**Text S1**

for

**“From molecular signal activation to locomotion: An integrated, multiscale analysis of cell motility on defined matrices”**

Amit Pathak and Sanjay Kumar

*Department of Bioengineering, University of California, Berkeley, CA, USA*

**Glossary of time-dependent variables and model constants**

**Table S1**: Independent time-dependent variables that comprise the dynamical system in our model (Eqs. 1-9 in the main manuscript).

| ***Symbol*** | ***Definition*** | ***Typical values*** | ***Model component*** |
| --- | --- | --- | --- |
| $\gamma$ | Rac activation level | 0 – 1 | Protrusion dynamics |
| $N$ | Number of cell-ECM adhesion bonds | 0 – 10^4^ | Adhesion dynamics |
| $N_{p}$ | Net growth rate of protrusion adhesions | 0 – 0.1 s^-1^ | Protrusion dynamics |
| $\rho$ | Rho activation level | 0 – 1 | Actomyosin contractility |
| $T$ | Contractile force due to stress fibers | 0 – 15 nN | Actomyosin contractility |
| $x_{C}$ | Displacement of cell centroid | 0 – 30 $\mu\text{m}$ | Cell body migration |

**Table S2**: Additional parameters used to describe the model.

| ***Symbol*** | ***Definition*** | ***Typical values*** | ***Model component*** |
| --- | --- | --- | --- |
| $\bar{f}$ | Concentration of FA proteins accumulated per receptor-ligand bond | 0 – 1 | Adhesion dynamics |
| $\delta$ | Bond stretch of receptor-ligand bonds | 0 – 20 nm | Adhesion dynamics |
|  | Stored potential energy per receptor-ligand bond | 0 – $6\times10^{-5}$ nN/μm | Adhesion dynamics |
|  | Growth rate of an adhesion cluster, due to formation of receptor-ligand bonds | 0 – 500 s^-1^ | Adhesion dynamics |
|  | Dissociation rate of an adhesion cluster, due to rupture of bonds | 0 – 5 s^-1^ | Adhesion dynamics |
| $\bar{N}^{(F,R)}$ | =$N^{(F,R)}/N_{o}$, normalized values of adhesion size at front and rear adhesions | 0 – 1 | Adhesion dynamics |
| $T_{C}$ | Contractile force generated by stress fibers | 0 – 15 nN | Actomyosin contractility |
| $T_{S}$ | Contractile force sustained by stress fibers | 0 – 15 nN | Actomyosin contractility |
| $\varepsilon$ | Deformation of cell-ECM adhesion bonds | 0 – 10 nm | Actomyosin contractility |
| $\bar{T}^{(F,R)}$ | =$T^{(F,R)}/T_{o}N_{o}$, normalized values of tension generated by the stress fibers attached at front and rear adhesions | 0 – 1 | Actomyosin contractility |
| $F_{P}$ | Net forward propulsive force | 0 – 15 nN | Cell body migration |
| $F_{CB}$ | Resistive drag force due to cell body adhesions | 0 – 5 nN | Cell body migration |
| $\bar{t}_{C}$ | Retraction period of the rear edge at steady state | 0 – 100 min | Cell body migration |
| $\bar{x}_{C}$ | Migration displacement of cell centroid per cycle | 0 – 10 $\mu\text{m}$ | Cell body migration |
| $v$ | Average migration speed = $\bar{x}_{C}$/$\bar{t}_{C}$ | 0 – 30 $\mu\text{m/hr}$ | Cell body migration |

**Table S3**: Rate constants and proportionality constants used in the model.

| ***Symbol*** | ***Definition*** | ***Value*** | ***Model component*** |
| --- | --- | --- | --- |
| $\gamma_{o}$ | Maximum Rac activation level in the rear | 0.1 | Adhesion dynamics |
| $N_{o}$ | Maximum number of bonds in an adhesion cluster | 10^4^ | Adhesion dynamics |
| $k_{RL}$ | Elasticity constant of the receptor-ligand bond | $0.15 \text{nN/μm}$ | Adhesion dynamics |
| $k_{S}$ | Elasticity constant of the cell-ECM bond | 0 – 0.15 $\text{nN/μm}$ | Adhesion dynamics |
| $\kappa$ | Normalized ECM stiffness = $k_{S}/k_{RL}$ | 0 – 1 | Adhesion dynamics |
| $k_{f}$ | Elasticity constant of the FA proteins | $k_{RL}/2$ | Adhesion dynamics |
| $\delta_{c}$ | Critical bond stretch of the receptor-ligand bond | $20 \text{nm}$ | Adhesion dynamics |
| $\lambda$ | Ligand density | 0 – 10^5^ | Adhesion dynamics |
| $\bar{\lambda}$ | ECM adhesivity = $\lambda/N_{o}$ | 0 – 10 | Adhesion dynamics |
| $r_{o}$ | Reference rate constant for receptor clustering | $0.05 \text{s}^{\text{-1}}$ | Adhesion dynamics |
| $r_{d}^{-}$ | Rate constant for receptor-ligand bond dissociation | $0.001 r_{o}$ | Adhesion dynamics |
| $r_{p}^{+}$ | Constant intrinsic rate of adhesion formation at the filopodia | $2r_{o}/N_{o}$ | Protrusion dynamics |
| $r_{p}^{-}$ | Rate constant for dissociation of protrusion adhesions due to membrane ruffles | $0.001r_{p}^{+}$ | Protrusion dynamics |
| $\eta$ | Kinetic energy of membrane ruffles | $\eta=k\tau/N_{o}$ | Protrusion dynamics |
| $c_{\gamma}$ | Proportionality constant for Rac activation relation with protrusion formation | $2\gamma_{o}=0.2$ | Protrusion dynamics |
| $T_{o}$ | Maximum contractile force generated per actin-myosin motor | $1.5 \text{pN}$ | Actomyosin contractility |
| $k_{CE}$ | Equivalent stiffness constant of the cell-ECM bond | $k_{RL}k_{S}/\left(k_{RL}+k_{S}\right)$ | Actomyosin contractility |
| $k_{SF}$ | Passive elasticity constant of the stress fibers | $0.002k_{RL}$ | Actomyosin contractility |
| $\rho_{o}$ | Minimum possible value of Rho activation level assigned at t=0 | 0.1 | Actomyosin contractility |
| $r_{t}^{+}$ | Rate constant for the forward rate of actomyosin contractility | 0.01 s^-1^ | Actomyosin contractility |
| $r_{t}^{-}$ | Rate constant for the backward rate of actomyosin contractility | $2\times10^{-7}$ s^-1^ | Actomyosin contractility |
| $c_{\rho}$ | Proportionality constant for Rho activation with rise in contractility | $0.2 \text{nN}^{-1}$ | Actomyosin contractility |
| $r_{x}$ | Rate constant for the dissociation of transient cell-ECM bonds dispersed over the cell body | $3r_{d}^{-}$ | Cell body migration |
| $\mu$ | Frictional constant due to cell-body adhesions | $2k_{RL}\delta_{c}^{2}/N_{o}$ | Cell body migration |
| $\xi$ | Cell polarization factor | $\leq1$ | Cell body migration |
